# Pains after Extraction of the Teeth

**Published:** 1896-10

**Authors:** 


ARTICLE V.

### PAINS AFTER EXTRACTION OF THE TEETH.

(From *Dental Office and Laboratory*.

This becomes sometimes so severe as to rend sufferer almost wild with pain. What will relieve in one instant seems useless in another, so that we give all the remedies which have been suggested from time to time, that they may be tried in the effort to allay the suffering:

Of nitro-glycerine take a single drop *in a half glass of cold water*. The nitro-glycerine should be a one per cent. solution.

Inhale two drops of amyl-nitrate for three or four seconds, carefully, and follow the inhalation by complete rest for five minutes.

Fletcher's carbolized resin, which is composed of resin, carbolic acid and chloroform. The resin may be dissolved in the chloroform to saturation in a half ounce vial, and ten drops of carbolic acid added. It is also an excellent styptic.

Chloroform one part, and tincture of pyrethum. The combination on cotton placed in the socket.

Cleanse out the socket with phenol-sodique, then apply, on a loosely rolled pellet of cotton, the following:

| | |
|---|---|
| Glazial carbolic acid, . | ʒ ij. |
| Liq. potasse, . . . | ℥j. |
| Water. . . . | ℥ vj. |

M. *Gorgas' Dental Medicine.*

| | |
|---|---|
| Alcohol (best) . . | ℥j. |
| Chloroform, . . . | ℥ ij. |
| Sulphuric ether, . . | ℥ ¾ |
| Gum camphor, . . . | ℥ ss. |
| Tinct. opium, . . | ʒj. |
| Oil cloves, . . . | ʒ ss. |

S.—Apply in the socket on a pledget of cotton.

*T. B. Welch.*

Camphor, . . . . ℨj.

Chloroform, . . ℨij.

M. Apply to socket on cotton.

Chloroform, tinct. aconite equal parts. Apply to socket on cotton.

Morphia, . . . gr. vj.
Tinct. aconite,
Chloroform,
Alcohol, . . . aa fl. ℥j.

M. Apply on cotton in the Socket.

Relief is often obtained by heat—a dry hop poultice held next the cheek.

Wipe out the socket with a swab of cotton wound around a match stick, so as to remove all blood clots, and syringe the socket well with hot water.

The septum in the socket next the adjoining tooth is sometimes fractured in the effort of extraction, and little spiculæ of bone remain within the socket, keeping up irritation. These should be removed, if large enough to be seized with the tweezers, or syringed out with hot water if too small. Suppress the hemorrhage as much as possible, and apply to the socket cotton moistened with phenated camphor.

Dr. E. Sjoberg, of Stockholm, offers the following: Among the various remedies which are used for quieting the after-pain of tooth extraction, with or without resection, there is scarcely anything found more reliable than phenylic acid. First try a warm solution, say 3.5 per cent.; inject it into the bottom of the alveolus with an ordinary-sized syringe. Should the pain continue take phenylic acid alone, and fill the alveolus with the help of a syringe, with a bent end. After a few seconds take away the superfluous fluid with a taper end made of blotting paper. Either the pure phenylic acid or the strong solution is used; a napkin, reaching the edges of the alveolus, must be pressed to both sides of the same in order to protect the surrounding parts from excoriation.

Menthol. crys., . . gr. v.
Tinct. aconite, . . . gtt. xx.
Chloroform, . , . q. s. ʒ ij.

Sig.—Apply on gum over the seat of trouble with a pad of bibulous paper.

*Ohio Dental Journal.*

Wash out socket by syringing with hot water, to which a few drops of carbolic acid are added, and see there are no loose spiculæ of bone to act as irritants, then introduce cotton saturated with

R.—Menthol, . . . ʒ j.

Chloral hydrate, . . . ʒ j.

Camphor gum, . . ʒ ss.

Alcohol, . . . . fl ℥ j.

When the pain partakes of a neuralgic character antikamnia in 3 to 5 grains does, phenacetine in 5 to 10 grain doses will generally give relief.

The face may be bathed with a liniment of

R.—Laudanum, . . . fl. ʒ ij.

Tinct. capsici, . . fl. ʒ ss.

Spt. camph., . . fl. ℥ ss.

Oil sassafras, . . fl. ʒ ij.

*Dr. I. H. Morgan, Salem, Va., in Busy Dentist.*

In reporting a case of twelve days' pain after extracting a tooth to relieve periostitis, Mr. Bennett says relief was finally and instantly obtained by passing a Paget's knife into the socket and cutting across the nerve.—*Journal British Dental Association.*

